# If not police, then who? Building a new workforce for community behavioral health crisis response

**DOI:** 10.3389/fpsyg.2025.1579787

**Published:** 2025-04-25

**Authors:** Amy C. Watson, Kellan McNally, Leah G. Pope, Michael T. Compton

**Affiliations:** ^1^School of Social Work, Wayne State University, Detroit, MI, United States; ^2^New York State Psychiatric Institute, New York, NY, United States; ^3^Division of Behavioral Health Services and Policy Research, Columbia University College of Physicians & Surgeons, New York, NY, United States

**Keywords:** alternative response, 911 diversion, behavioral health crisis, mobile crisis intervention, workforce development, community response

## Abstract

**Introduction:**

Communities across the United States and elsewhere are working to implement alternatives to law enforcement as primary responders to behavioral health crises. These efforts can only be successful if there is a skilled workforce prepared to take on this role. We argue that this workforce must be developed, and propose a new, credentialed Community Behavioral Health Crisis Responder (CBHCR) role.

**Methods:**

Guided by a 13-member advisory board with expertise across behavioral health, crisis services, and law enforcement, we conducted a literature review, key informant interviews, and focus groups to identify the foundational values, competencies, and skills for this proposed role.

**Results:**

Interview and focus group participants discussed desired characteristics of CBHCRs and emphasized values such as cultural humility, a nonjudgmental approach, and the importance of lived experience broadly defined. Competencies and skills included engagement and communication strategies that enhance safety and trust, suicide prevention, conflict resolution, and situational awareness. Participants highlighted the need to train CBHCRs to provide compassionate, trauma-informed crisis intervention, de-escalation, support, and connection to needed resources. In conjunction with our advisory board and external experts, we used the findings to iteratively refine the values, competencies, and skills of CBHCRs.

**Discussion:**

We discuss the next steps in creating this new, skilled and credentialed crisis response workforce.

## Introduction

In the aftermath of the murder of George Floyd by Minneapolis police officer Derek Chauvin and the nationwide protests that followed, public support has grown for alternatives to police as first responders to mental/behavioral health related calls for service. Furthermore, in its investigations in several cities, the United States Department of Justice Civil Rights Division has identified the reliance on police as sole responders to behavioral health issues as a potential violation of the Americans with Disabilities Act (United States Department of Justice Civil Rights Division, 2023a, 2023b, 2024, 2025). As a result, communities across the country are creating (or exploring) alternative response teams capable of responding to 911 calls for service related to behavioral health and other social service needs. Staffing of these teams varies and may include clinicians, emergency medical technicians (EMTs), peer support specialists, and others with lived experience and community knowledge (Council of State Governments Justice Center, 2021). Information on how these teams are being trained is limited; however, a Reach Out Response Network report suggests programs typically develop training in-house (Reach Out Response Network, 2021).

During this same period, the COVID-19 pandemic prompted public concern about mental health and accelerated federal and state efforts to develop comprehensive mental health crisis services, including the development or expansion of mobile crisis response capacity (NRI, 2024a) and a push to divert some 911 calls for service to the mental health crisis system via 988 and mobile crisis teams. However, as a recent NRI (2024b) State Profiles report indicates, many states (90 percent) have behavioral health crisis workforce shortages. In terms of mobile crisis team staffing, states report the greatest shortages being social workers (MSWs), other licensed clinicians, and peer support specialists. To address these shortages, states are implementing recruitment efforts, increasing pay, offering educational grants, changing educational requirements, and developing crisis certification programs (NRI, 2024a). While these efforts are promising and needed, they are being implemented in the absence of established and formalized values, competencies, skills, and training standards for behavioral health crisis response in the community. States and agencies have traditionally relied on specific educational and licensure requirements when defining qualifications for behavioral health crisis responders. However, such traditional requirements may not reflect the actual skills (or interest) needed for crisis response, particularly response to 911 behavioral health crisis calls.

Thus, a critical step in advancing efforts to reduce the role of law enforcement in behavioral health crisis response is the development of a workforce that is motivated and prepared to serve as primary first responders to people experiencing mental health crises. While current SAMHSA guidelines (Substance Abuse and Mental Health Services Administration, 2025a) suggest that teams including master’s-level licensed or credentialed clinicians should respond, the current workforce does not have the capacity to meet current demands, is not prepared for this first responder role, and in many cases, citing safety concerns, does not want to go into the community without police. We sought to address the question: How can we develop a diverse, non-law enforcement workforce to respond to mental health crisis calls that is rapidly scalable?

Workforce development in the traditional sense, such as providing existing professionals more training or expanding numbers in existing professional roles, is unlikely to adequately address the need for skilled crisis responders prepared to work in diverse communities. We have written about the potential for a new first responder professional—a Community Behavioral Health Crisis Responder (CBHCR)—that is trained in crisis intervention, is equipped with the skills to safely respond to most mental health crises without law enforcement and has the judgement to request law enforcement assistance when appropriate (Carroll et al., 2021; Watson et al., 2021). The CBHCR would be in effect, akin to an EMT or paramedic, but for behavioral health crises rather than for medical emergencies.

In this article, we provide an overview of the work we have done to develop a foundation of values, competencies, and skills for these new professionals that could be hired to work on community/alternative response or mobile crisis teams. The first stage of the work involved convening a 13-member advisory board and reviewing the literature on crisis response professionals. We then conducted key informant interviews with professionals with content expertise related to mobile crisis response, alternative/community response, peer crisis services, law enforcement, and emergency medical services; and focus groups with front line crisis/community response workers, people that have used crisis services, and family members of those who have used crisis services. In collaboration with an advisory board, we used the findings to identify the values, competencies, and skills of CBHCRs.

### The advisory board

Our work was guided by an advisory board comprised of 13 members with subject matter expertise related to behavioral health crisis services, youth crisis services, peer crisis services, psychiatry, harm reduction, alternative response, law enforcement, mental health policy, and advocacy. Throughout the project, we met quarterly with the board to get their feedback on our plans and progress, and to discuss their feedback on several rounds of revisions to the draft CBHCR values, competencies, and skills. Initially, we had set out to identify the competencies and skills needed for this new workforce. Early in the project, the advisory board suggested, and we agreed, that we should also consider CBHCR professional values. As a result, we included questions about values in our interview and focus group guides and examined themes related to values that emerged from our data.

### The literature review

We searched academic and gray literature for research on skills for professionals working in crisis and related services and the experiences of people who have utilized crisis services. This included literature on co-responder clinicians, crisis line clinicians, alternative/community response team members, law enforcement officers, and violence prevention specialists. We found consistent themes across different professional groups and crisis settings. Overall, the literature indicates responders need to be able to recognize signs and symptoms of mental illness and substance use crises and have skills to approach and engage individuals experiencing crisis in a compassionate, nonjudgmental manner (Ghelani, 2021; Holgersen et al., 2022; Lavoie, 2018; Sands et al., 2013; Thompson et al., 2021; Wheeler et al., 2015; Xanthopoulou Thomas and Dooley, 2022). De-escalation skills, often defined in terms of active listening, validation, reflective statements, body language, and tone of voice are consistently mentioned, as are conflict resolution and knowledge of community resources (Sands et al., 2013; Schleiffer and van Lier, 2022; Todak, 2017; Xanthopoulou Thomas and Dooley, 2022). Safety skills noted include situational awareness and many of the skills listed as important to effective approach and engagement (e.g., staying calm, active listening, being nonjudgmental, providing choices) (Fischer et al., 2020; Reach Out Response Network, 2021; Thompson et al., 2021; Weisman and Lamberti, 2002; Weisman, 2011).

The literature specific to mental health professionals and alternative responders highlights the need for suicide prevention and intervention skills, understanding and use of trauma-informed skills, cultural sensitivity and understanding of race equity, motivational interviewing, harm reduction, conflict resolution/mediation skills, and basic medical skills (Ghelani, 2021; Reach Out Response Network, 2021; Sands Elsom et al., 2016; Thompson et al., 2021). Professional boundaries and management of secondary trauma are noted in the literature on violence prevention specialists and alternative responders (Fischer et al., 2020; Reach Out Response Network, 2021; Thompson et al., 2021).

The limited literature on the experiences of people who have used crisis services highlights the qualities of crisis care that people and their family members desire. These include compassionate, supportive, respectful, and kind response; involvement in decision making; inclusion/acknowledgment of family members/carers; and attention to basic needs and comfort (Holgersen et al., 2022; Lavoie, 2018; Sands Elsom et al., 2016; Thomas et al., 2018; Wheeler et al., 2015; Xanthopoulou Thomas and Dooley, 2022).

## Methods and materials

### Key informants interviews

Key informants (KI) were recruited for interviews through our professional networks. We conducted 11 interviews via Zoom with professionals representing law enforcement (*n* = 1), emergency medical services (*n* = 2), crisis services/mobile crisis (*n* = 3), community/alternative response (*n* = 3), and peer crisis services (*n* = 2). We asked each KI to share their perspectives on effective community behavioral health crisis response. Interview guides included specific probes asking them to discuss the skills needed and not needed, safety concerns and safety protocols, training and formal education, characteristics of people well suited for the work, where CBHCRs should be housed, and policy and implementation issues.

### Focus groups

To gain the perspectives of key stakeholders (individuals who provide and/or use crisis services), we conducted a total of 13 focus groups involving 60 individuals, with professionals working in mobile crisis and community response (3, *n* = 15), adults with lived experiences of mental illness and crisis services (5, *n* = 27), young adults with lived experiences of mental illness and crisis services (2, *n* = 7) and family members (3, *n* = 11). To recruit professionals working in crisis/community response, we reached out to national and local organizations working in this space. Adults and young adults with lived experiences were recruited via peer support networks, NAMI groups, youth networks, and Project LETS. Family members were recruited via NAMI and other professional contacts. Interested individuals who contacted the project team by email were provided with information about the project, and if interested in participating, scheduled for a 90-min focus group session conducted via Zoom. Participants received a $50 Amazon electronic gift card for their participation.

Three focus group guides were developed. Crisis response professionals were asked to discuss the types of calls they respond to; the skills they need to do their job; challenges they encounter; and the values, qualifications, characteristics, training, and skills that community/crisis responders should have. People with lived experience and family members were asked to discuss what a behavioral health crisis is like for them (or their loved one); what is helpful and not helpful during a crisis; what makes them feel safe or unsafe; and a set of questions parallel to those asked of community/crisis responders about the values, characteristics, and qualifications that community crisis responders should have.

We made a concerted effort to distribute the recruitment information to a diverse range of constituents. We did not ask participants to complete a formal demographics survey. Demographics reported in Table 1 are based on indicated preferred pronouns and race/ethnicity stated in discussion. While the majority of those who participated identified as women (72%), participants were more diverse in terms of race/ethnicity, particularly for the group of participants who reported lived experience of mental illness.

**Table 1:** Focus group demographics.

Group	Gender	Race/Ethnicity
	Man (%)	Woman (%)	Trans-gender (%)	Non binary (%)	Un-known (%)	Black (%)	White (%)	AAPI* (%)	LatinX (%)	Un-known (%)
Lived experience	4 (11.8)	25 (73.5)	2 (5.9)	2 (5.9)	1 (2.9)	8 (23.5)	14(41.2)	2 (5.9)	8 (23.5)	2 (5.9)
Family	3 (27.3)	8 (72.7)				2 (18.2)	9 (81.8)			
Responder	5 (33.3)	10 (66.7)				2 (13.3)	10 (66.7)		3 (20.0)	
Total	12 (20.0)	43 (71.7)	2 (3.3)	2 (3.3)	1 (1.7)	12 (20.0)	33 (55.0)	2 (3.3)	11 (18.3)	2 (3.3)

*Asian American and Pacific Islander.

All study activities were determined exempt (KI interviews) or approved (focus groups) by the Institutional Review Board at the first author’s home institution.

### Analysis approach

All interviews and focus groups were conducted via Zoom and were recorded and transcribed for analysis. A thematic approach was used for analysis, with codes developed inductively based on text in the transcripts and deductively based on the research questions (Braun and Clarke, 2006). Coding was completed using Dedoose software. The first and third author and two research assistants read the transcripts, identified codes, grouped the codes into categories to develop themes, and drafted memos to identify the themes reported here. At least two team members read and coded each transcript, and discrepancies were resolved by the team.

#### Drafting of the values, competencies, and skills of CBHCRs

To create the initial draft of CBHCR values, we consulted our advisory board and reviewed interview and focus group findings for values-related themes. To consolidate the findings related to competencies and skills, we created a spreadsheet listing competencies and skills identified in the literature review for different crisis professionals and by people who have used crisis services and added the competencies and skills identified in our analysis of interview and focus group data. We met weekly as a team and reviewed the competency categories and skill groupings and discussed discrepancies among team members and overlap among categories. From this spreadsheet, we created a draft competencies and skills document. Both draft documents were sent to the advisory board for review in advance of a quarterly meeting. At the quarterly meeting, the members of the board provided initial feedback. The feedback was incorporated and revised drafts were then distributed to the board via email with a request for written feedback. This subsequent round of feedback was used to further revise the drafts before they were sent to several KIs who had indicated an interest in reviewing, as well as several additional content experts in the field. This feedback was incorporated into an almost final draft shared with the advisory board and discussed at a quarterly meeting. Final edits were made following that meeting to produce the Values (see Table 2) and Competencies and Skills (see Tables 3–5).

**Table 2:** Core values of community behavioral health crisis responders (CBHCRs).

DIGNITY: CBHCRs respect the inherent dignity and worth of every person. They demonstrate this by responding to community members nonjudgmentally and with compassion. Through their actions, as well as ongoing learning and self-reflection, CBHCRs strive to consistently recognize the wholeness and humanity of every person they serve.
RELATIONSHIP: CBHCRs recognize the central importance of human relationships and are committed to providing care in a compassionate and trustworthy manner that supports the need of those contacted to feel safe as they make decisions about engaging in care. This requires conveying genuine respect for the person, focusing on needs and preferences as defined by that person, and demonstrating clear, honest, and transparent communication.
COLLABORATION: CBHCRs view their role as collaborative, and work to support people in expressing their personal goals and preferences, and in harnessing their own strengths and resources.
AUTONOMY: CBHCRs are committed to forms of support, interventions, and approaches that enhance the autonomy of the people they serve.
SUPPORT SYSTEM: CBHCRs recognize the importance of the person’s family/chosen family/social support system. As they work to support people in crisis, CBHCRs demonstrate an awareness of these dynamics, and strive to support the relevant needs of others in those relationships/networks. At times, this work includes helping people who have been isolated forge new connections to communities of support, if they want them.
INTEGRITY: CBHCRs act with integrity. They are committed, honest, trustworthy, and reliable. They act conscientiously, consistently, and in accordance with the other stated values.
ADVOCACY: CBHCRs stay informed of and are ready to advocate for resources in the communities where they work. Their advocacy addresses barriers and gaps in services that impact people at individual and systemic levels, and work to bring about change by directly communicating the needs of those who they support to providers and policymakers
TRAUMA-INFORMED: CBHCRs have an ongoing commitment to evolving their knowledge about trauma, harm reduction, and histories of oppression in all forms.
LIVED EXPERIENCE: CBHCRs recognize the value of lived experiences in providing effective responses. On teams where they work, CBHCRs listen, learn from, and partner with those who have experiences and carry understandings that differ from their own.

**Table 3:** Competencies and skills: relationship-building and communication.

RELATIONSHIP-BUILDING COMPETENCIES
Self-awareness: CBHCRs possess and practice self-awareness that allows them to forge trusting and collaborative connections. By understanding their personal biases and monitoring themselves for emotional reactions, CBHCRs provide support based on the needs and preferences of the people they serve. They care for themselves and regulate their own emotions and maintain appropriate boundaries. *Example Knowledge/Skill Areas* Emotional self-regulation Flexibility in thinking and decision making Meeting people where they are at Use of self-disclosure appropriately for the benefit of the person served
Collaboration: CBHCRs engage with the people they support in a shared process of planning for and accessing resources. In this collaborative process, CBHCRs learn from the person they are serving what their unmet needs are, provide information on available resources, and assist the person in accessing desired resources. *Example Knowledge/Skill Areas* Relationship building Meeting people where they are at Collaborative/shared action
Unconditional positive regard: CBHCRs can be relied on to consistently show respect, genuine concern, and a desire to help. By maintaining a compassionate approach, trust is built as CBHCRs learn from each person what they need and what their goals are. CBHCRs understand that even if a person’s behavior seems unhealthy, it is directed toward getting a need met. The goal of CBHCRs is to help people find safer and healthier ways to meet their needs. *Example Knowledge/Skill Areas* Respect, nonjudgment, and compassion Genuinity Showing concern about and providing for person’s comfort and wellbeing
Engagement: CBHCRs communicate their genuine desire to help. They recognize and validate harm caused by the structural conditions that people face. They meet people where they are at and are responsive to their preferences and expressed needs. *Example Knowledge/Skill Areas* Communicating a desire to help Providing emotional support/validation Addressing immediate needs

COMMUNICATION COMPETENCIES
Active listening: CBHCRs possess an ability to listen, acknowledge, and validate, making purposeful use of personal experiences to facilitate understanding and connection. They communicate honestly about the availability of resources and support people to make informed choices. *Example Knowledge/Skill Areas* Providing emotional support/validation Openly and honestly discussing the availability of resources
Verbal and nonverbal communication: CBHCRs know how to adjust the tone and pitch of their voices to build trust and clearly convey information. Technical skills may also reflect the use of communication technologies, language resources, and translation services that increase access to information and support. CBHCRs use non-verbal communication to increase feelings of safety and support. *Example Knowledge/Skill Areas* Vocal modulation Communication technologies, non-English and non-hearing language resources Non-verbal communication
Culturally responsive communication: CBHCRs tailor their communication to engage individuals from diverse experiences and backgrounds while maintaining an openness to learning. They avoid jargon and other professional scripts. This allows for discussions across differing perceptions of resources that may be requested and recommended. *Example Knowledge/Skill Areas* Flexibility in thinking, decision making, and communication Age-related preferences Developmental and learning differences Transcultural skills

**Table 4:** Competencies and skills: knowledge-based competencies and skills.

Mental/behavioral health: CBHCRs possess the introductory-level skills and knowledge required to recognize signs of behavioral health distress, including those stemming from mental health and substance-related conditions. This knowledge allows CBHCRs to evaluate acuity for individuals experiencing a behavioral health crisis and an ability to triage cases to the appropriate and least-restrictive level of care. Understanding of common symptoms and crisis states allows CBHCRs to foster trust and facilitate connection without alienating individuals or contributing to their distress. Competency in this area includes the ability to identify common medications used in psychiatric and substance use disorder treatment and changes in behavior and/or physical health that indicate adverse reactions. *Example Knowledge/Skill Areas* Signs and symptoms of psychiatric, substance use, and co-occurring disorders Knowledge of common medications Evaluation of symptom acuity and appropriate level of care Seeking support/supervision when needed
Cultural humility and anti-oppressive practice: CBHCRs understand the effects of racism and other forms of institutionalized oppression. They engage in cultural humility and anti-racist practices while making timely use of team-based and non-English language resources and ensuring services are provided confidentially within a trauma-informed framework. *Example Knowledge/Skill Areas* Anti-racist/Anti-oppressive practice Disability justice Cultural humility Trauma-informed care
Physical health: CBHCRs are alert to the signs of physical health conditions (e.g., hypoglycemia, post-seizure state, delirium) that can mimic psychiatric distress as well as other basic medical issues that may warrant EMS/medical attention. CBHCRs recognize physical health concerns related to the use of substances, including use patterns that put people at risk for life threatening withdrawal and overdose. They provide emergency intervention to reverse overdose with medications when appropriate. CBHCRs employ harm-reduction strategies to minimize harmful impacts of high-risk behaviors and engage emergency medical services when needed. *Example Knowledge/Skill Areas* Alert to signs indicating the need to engage EMS/medical services Recognition of signs of overdose and administration of emergency overdose reversal medications Harm reduction approaches Basic CPR/first aid
Safety assessment and intervention: CBHCRs evaluate risks and strengths in complex situations. Skills in this domain include an ability to engage in conversations related to basic suicide/self-harm and violence risk assessment and intervention. Other assessment capabilities may be driven by local contexts that increase the demand for CBHCRs with training around specific issues like gang violence and human trafficking. *Example Knowledge/Skill Areas* Suicide/self-harm risk assessment and intervention Violence risk assessment and intervention
Evidence-based interventions: CBHCRs have training in structured, evidence-based approaches such as motivational interviewing, brief addiction counseling, and collaborative problem-solving. They use these skills in ways that create options for people as they learn about and engage with services. *Example Knowledge/Skill Areas* Motivational interviewing Brief addiction counseling Collaborative problem solving Safety planning
Documentation and privacy practices: CBHCRs demonstrate proficiency in timely documentation, which is essential for compliance, accurate record-keeping, and team-based service delivery. CBHCRs understand regulations related to information sharing and confidentiality, especially as it relates to sharing protected health information with law enforcement or others. *Example Knowledge/Skill Areas* Federal and state regulatory and legal requirements Documentation practices HIPAA/privacy regulations

**Table 5:** Competencies and skills: safety-related and additional competencies and skills.

**SAFETY-RELATED COMPETENCIES**
Managing the physical environment: By assessing and managing the physical environments where services occur, CBHCRs maintain their own and others’ safety. CBHCRs remain prepared to de-escalate and disengage when necessary. *Example Knowledge/Skill Areas* Spatial/physical safety considerations Environment management/physical space Self-regulation
Preparation: A CBHCR’s preparation before entering an encounter lays the groundwork for effective and safe service. Preliminary and ongoing assessment of resources and risks includes making both individual and environmental considerations. This entails assessing for potential weapons and other risks in the response environment and developing plans to manage those risks. This planning may involve collaboration with the person to be served (for example, asking the person to secure weapons or pets in advance of arrival or in the early stages of the crisis response). *Example Knowledge/Skill Areas* Gathering information Planning for safety Collaborating with the person to be served. Dynamic risk assessment
Crisis de-escalation and maintaining safety: CBHCRs are equipped to actively maintain safety and possess skills that include clear limit-setting, crisis de-escalation skills, disengagement when needed, and basic nonviolent self-defense. These active skills enable them to respond to challenges without compromising their well-being or the safety of those whom they assist. *Example Knowledge/Skill Areas* Limit-setting/boundaries Crisis de-escalation (verbal de-escalation skills, body language, use of space) Disengagement Nonviolent self-defense

ADDITIONAL COMPETENCIES
Resource navigation and advocacy: CBHCRs utilize knowledge of local resources, relationships with other service providers, and advocacy skills to help people find needed resources. CBHCRs are adept in basic case management and able to support people as they navigate complex and social systems such as those pertaining to housing, physical health and behavioral health treatment, legal needs, food security, and child welfare. *Example Knowledge/Skill Areas* Knowledge of local resources Basic housing system knowledge Basic legal system knowledge Family/social network engagement Advocacy Case management
Self-care: CBHCRs self-advocate, seek supervision, and cope with the potential stress their work can pose to their health. This includes considering aspects of their work that may trigger personal reactions (e.g., sexual assault, domestic violence), and working with their supervisors to manage these situations. Self-care also involves setting boundaries and finding work-life balance to help prevent burnout and compassion fatigue. *Example Knowledge/Skill Areas* Routine practice of stress management and effective coping skills Boundary-setting Utilizing supervision

## Findings

We present themes related to the values, competencies, and skills needed for effective community behavioral health crisis work that emerged from key informant interviews and focus groups together given overlap in the content. We start by discussing themes related to the desired characteristics and qualifications of crisis responders as we feel they provided useful information and context for our subsequent development of CBHCR values, competencies, and skills. Although there was substantial agreement between and across key informants and focus group participants on many topics, we also noted several issues on which perspectives varied.

### Personal and interpersonal characteristics

Participants discussed the importance of responders having a deep capacity for compassion and empathy, the right motives for doing the work, and the ability to be nonjudgemental.

*I think they really need to walk in, like you said, nonjudgmental, and showing that empathy. Not, you know, kind of laughing inwardly or being afraid... People can sense that stuff, so I think that belief system really has to be inside of them.* [family member].

Participants also indicated that crisis responders need to be adaptable and able to work under pressure, as well as able to keep themselves calm, self-regulate, and manage their reactions to intense situations. Further, they need to be comfortable in physically and emotionally uncomfortable situations and be resilient, as the work is difficult at times. As one KI stated, *“I think that’s truly the thing— it’s like people who are comfortable in uncomfortable situations are really, really good.”* Finally, participants centered the importance of diverse lived experiences as a core characteristic of crisis responders.

*But, you know, in my view, the more that people, you know, for lack of a better word have had tough experiences, you know, the... sort of the more they have to bring to the table, I think, in terms of a kind of compassion.* [KI].

While participants talked about these characteristics in terms of what to look for when hiring crisis responders, they also discussed how training and supervision could support the development or enhancement of some of these qualities.

### Qualifications and education

Views on educational and professional qualifications reflected participants’ conceptions of the work that crisis responders were expected to perform. For example, participants who saw crisis intervention as a critical step in the process of linking people to acute psychiatric and hospital-based services regarded master’s-level preparation and licensure as a needed qualification for completing suicide-risk assessments, level of care determinations, and involuntary commitment petitions. Other participants considered crisis response in terms of providing support and connection to resources and framed lived experience (broadly defined), rather than formal education, as central to preparation for working as a crisis responder. From that perspective, they pointed to a deep and genuine drive to serve others that came from personal experiences with the hardships and systems that clients dealt with and struggled to overcome. Across this range of ideas about qualifications, some participants noted the importance of crisis teams made up of people with diverse backgrounds and education levels.

*So, I have on my team individuals who just completed their GED all the way to people who have master’s degrees…I think to be an effective crisis intervention specialist, it requires more a goodness of personality and willingness to learn than it does traditional academic acumen* [KI].

Several KIs with master’s degrees and clinical licensure noted that the skills they use as crisis services providers are not skills they learned in graduate school.

### Values

Many of the values needed for community behavioral health crisis response are reflected in the characteristics, competencies, and skills described by participants. When asked directly about values, participants discussed an overall commitment to an anti-oppressive and person-centered practice that is non-hierarchical, empowering, and collaborative. Moreover, participants felt crisis responders should be trustworthy and transparent in their conduct and should show unconditional positive regard for the individuals, families, and other caregivers who they encounter.

*But we want that eagerness to learn and to be more self-aware and to understand your own biases. We’re going into people’s homes, and we are stepping into, you know, their space, their culture, their needs, and so we really want people to have an awareness of their own privilege -- just being in this position, privileges that they carry with them, coming into, you know, people’s crisis situations, and being able to respond appropriately and ethically, and equitably.* [responder].

*I think something that helps is when the responders see you as a person with a mental illness; as a person first. They do not treat you like a diagnosis; they do not treat you like you are a problem; they do not treat you like you are a burden.* [lived experience].

*And I think part of, like, meeting the clients where they are at is also, like, letting them know that they are the ones in control of their recovery. Like, we are not in control of their recovery, we are just helping facilitate that.* [responder].

### Competencies and skills

Participants discussed a variety of skills that we have grouped into three broad and sometimes overlapping competency categories for the purpose of reporting our findings. The first category pertains to communication and engagement competencies that support responders’ ability to connect and form an alliance with the person in crisis. The second category covers knowledge domains and skills, some of which overlap or support communication and engagement, and includes basic assessment and intervention frameworks. Given concerns about safety when police are not part of the response, we look at safety skills separately as the third category, although there is overlap with the other two categories.

#### Communication and engagement

Both KIs and focus group participants discussed the importance of competencies and skills related to communication, engagement, rapport, and trust building in supporting responders’ ability to form genuine and supportive connections with individuals in distress. Participants provided examples of these skills that included active listening, controlling tone, speaking respectfully, being transparent, and centering the expressed needs of the person experiencing crisis.

*Being with someone, like, with a calm voice, patient. Just having someone where I’m not alone. I’m not alone, but I have to have that initial, like, trust—like, that comfort when they are introducing themselves, that initial reaction, like, “Hey, this is someone coming at me from a good place.” You know? Just that comforting, peaceful voice, you know? You can feel that rapport, like, immediately with someone, not someone coming in, like, all clinical with all their paperwork out ready to get signed and pushing their goals on you.* [lived experience].

*What I find to be helpful is just, like, transparency and understanding… To take the listening ear, and to be, to be patient with the person who is in crisis and, also, with the family.* [family member].

*Yeah, we do a lot of meeting people where they are. Not necessarily where we want them to be, but exactly where they are. If you are having the worst day of your life, let us meet there. Let us not meet at a promise, or everything is gonna be okay, or — let us just meet where you are in that second. And let us move from there. If you are standing up, I’ll stand with you. If you are sitting down, I’ll sit with you. If you are crying, I’ll find a way to also tap into that, as well, to show you that I might not cry, but I’m there for you. I’m feeling you, I’m 100% there with you in every second of it.* [responder].

They discussed skills related to the ability to approach a person in a nonjudgmental, compassionate manner that helps them feel safe to engage and form an alliance.

*The other kind of core aspect to this is really around alliance*—*you know, being able to form an alliance with somebody, so that you come along to their side and suspend judgment or other things so that they truly feel, like, seen by you; they feel heard by you; they feel that their distress or their intensity is validated, rather than dismissed or rather than kind of, you know, pushed away*. [KI].

Focus group participants described a range of practices and skills to enhance people’s feelings of emotional safety and willingness to engage during crisis responses. For some, this meant maintaining a calm and warm demeanor, not rushing the process, asking permission, and giving undivided attention. For participants with lived experience, feelings of safety were diminished when crisis services felt designed to take away a person’s agency, were provided in police stations or hospital settings, and when crisis responders did not share identities of the people they served.

*I think, like, things that help to kind of preserve your sense of agency are really valuable. So, for everything that exists in the system, it seems like the goal is to take away your agency, and for me, that is, I cannot imagine how that would be grounding or healing or reassuring for anyone at all. And, so, I think being able to make decisions, and people trust the things I’m saying, and, like, trust that I can still say what I need. It feels like there’s some aspect of people being in crisis that leads people to be a little paternalistic towards them often and not really believe what they are saying. So, I think when people trust me, believe me, and that, like, still want to protect my agency and feeling of choice, you know, I think that’s really valuable and makes me feel safe and healthy.* [lived experience].

KIs also discussed the importance of having skills to provide a calm, compassionate approach to suicide intervention:

*The ability to, you know, enter into conversations around suicidal intensity, thoughts of wanting to die, etc., and be able to kind of hold that conversation and the spirit of respect and compassion is a core thing that many people do not have, and that historically in our systems… conversations related to suicide are treated, you know—have been historically treated as a sort of a red flag area that calls to mind a different kind of response. And so, people tend to shift out of the compassionate listening mode into something that feels more, you know—that might be called risk assessment but often for people feels a little bit more like grilling or testing, and feels driven by... again, by fear rather than compassion*. [KI].

#### Knowledge domains and skills

Participants spoke about general domains of knowledge and skills that support effective response. They indicated crisis responders should know about local services and populations in the communities where they work, have basic de-escalation skills, be able to recognize signs and symptoms of mental illness and substance use disorders, have risk assessment and triage skills, and understand how to demonstrate cultural competence/humility. Some participants spoke about the importance of specific frameworks or models such as motivational interviewing, trauma-informed care, and basic first aid.

De-escalation skills were described as including using time, distance, space, body language, tone of voice, and active listening to help the person feel safe. As one KI stated, *“They’re using their voice, their behavior, their total presentation with this person to feel safe. And they allow themselves to come down from ‘Red Alert’ stage, right?”* Participants discussed trauma-informed skills in terms of understanding that a person’s behavior may be shaped by past trauma (including trauma related to the mental health system) and using specific strategies to approach a situation in a trauma-informed way.

*I think you know the trauma literature has a nice way of putting it, which is, like, if when we see somebody to kind of ask, you know, what’s happening? Or what has happened to you? ... as opposed to, you know, what’s wrong with you?* [KI].

Specific motivational interviewing skills were discussed, along with the importance of internalizing the pillars of the approach.

*Huge; important. Supportive, validating listening; active listening; open-ended questions—those are things that you can teach in a classroom and roleplay those things out. And it’s funny, when you, like, truly listen to somebody, how quickly they calm down because they feel heard*. [KI].

#### Safety skills

We asked participants specifically about the safety skills that CBHCRs need in order to respond in the community without law enforcement. Responses focused on the approach and quality of the interaction, de-escalation skills, managing the physical environment, and specific operational strategies. Participants indicated the way the CBHCR approaches the situation, and the quality of the interaction, impacts safety for all involved. Specific strategies include maintaining a calm tone of voice and nonjudgmental attitude, being non-reactive, having flexibility to adjust approach, giving choice, gaining consent, and addressing basic needs and comfort.

*We never want to do things without them being involved and try to… keep them in charge of their life as much as possible, rather than stepping in and telling them what to do and taking over. Because the more that we can help them to feel in control, the better it will be for them in the long run, and the more that they’ll be able to make decisions around what will work for them. But if we start snatching away all their power, it just will escalate things*. [KI].

Participants also noted the importance of skills to de-escalate potentially volatile situations.

*So, one of the trainings that we have all of our staff go through is a nonviolent crisis intervention training, which will hopefully, you know, give us the tools to de-escalate when clients start to maybe get a little bit more aggressive.* [responder].

They indicated that managing the physical environment requires providing the person time/space to self-regulate.

*They need space to let that off. And so, some core skills around creating that are not just about, you know, sort of removing things from the room, but creating space for people to down-regulate themselves. Sometimes, you know, that means just making sure that others do not feel trapped; they do not feel contained.* [KI].

It also requires situational awareness, staying at an appropriate distance, knowing routes to exits, backing away if needed, and basic self-defense.

Finally, participants discussed specific safety strategies such as gathering information before arriving on scene, meeting with the person outside of their home (while also attending to privacy), always responding in pairs, staging police nearby for higher risk encounters, and using radios or other mechanisms to do status checks on team members. While some participants suggested operational strategies such as linking responders to police radio systems with panic buttons, wearing ballistic vests, and having police first “clear the scene,” others indicated safety is enhanced by removing features that they associated more with police. They believed CBHCRs should never use physical force, should always be unarmed, and should not dress in a militarized manner.

### Finalized values, competencies, and skills of CBHCRs

The findings above were used to develop the final list of Values, Competencies and Skills. Core Values of CBHCRs are displayed in Table 2. The values of *Dignity* and *Relationship* reflect the importance of compassion, nonjudgement, and recognizing the humanity of the people CBHCRs serve. *Relationship* recognizes the primacy of connection and reflects the need for CBHCRs to be genuine, trustworthy and transparent so that those served can feel safe in engaging in care. Both *Collaboration* and *Autonomy* recognize the agency of the person being served and the CBHCR’s role as supporting them in expressing their preferences and making decisions about their own care. The *Support System* value recognizes the importance of the person’s chosen support system and their preferences for support system involvement in their care. This may mean including family members or friends. It may also mean assisting the person in reconnecting with their support system or connecting with new communities of support. *Integrity* is crucial to providing ethical, transparent and trustworthy care. *Advocacy* reflects the need for CBHCRs to be advocates at heart, addressing service gaps and inequities at both the individual client and community levels. CBHCRs must also be *Trauma-Informed*, which requires a commitment to lifelong learning and self-reflection. Finally, CBHCRs recognize the value of diverse *Lived Experience* and expertise, including that of their team members and of the people they serve.

Competencies and skills, displayed in Tables 3–5, are grouped into four categories: (1) Relationship Building and Communication, (2) Knowledge-Based, (3) Safety Related, and (4) Additional Competencies. The first category (See Table 3) encompasses the competencies and skills prioritized by people with lived experience and family members, as well as many of the key informants and crisis responders. *Relationship Building* includes competencies of self-awareness, collaboration, unconditional positive regard, and engagement. Skills in these competencies include emotional self-regulation, meeting people where they are at, showing concern and providing for the person’s comfort, addressing immediate needs and providing emotional support and validation. *Communication* competencies include active listening, vocal modulation, non-verbal communication, and adjusting communication style to accommodate cultural preferences and developmental and learning differences. Overall, the competencies and skills in this category support the development of rapport and emotional safety necessary for effective collaboration with the person experiencing a crisis.

*Knowledge-Based* competencies provide CBHCRs with tools to effectively provide support and intervention to the people they serve (see Table 4). This includes basic knowledge of mental health and substance related conditions and the skills to assess acuity and triage to the least restrictive level of care, and to conduct risk assessments (suicide/self-harm and violence) and collaboratively safety plan. CBHCRs are trained in evidence-based interventions such as motivational interviewing, brief addiction counseling, collaborative problem solving, and safety planning. They are also trained in recognizing medical issues that may require immediate medical attention, identifying signs of withdrawal and overdose, administration of emergency overdose reversal medications, applying CPR and basic first aid, and using harm reduction approaches. The competency of cultural humility and anti-oppressive practice requires that CBHCRs understand the effects of racism and other forms of institutionalized oppression and approach their work with a trauma-informed lens. Finally, CBHCRs understand and work within regulatory and legal requirements. This includes understanding regulations related to confidentiality, information sharing, and mandated reporting. This also includes maintaining timely and accurate documentation, which is essential for compliance and team-based service.

While skills related to *Relationship Building* and *Communication* are critical to safely providing crisis support, *Safety-Related* competencies include managing the physical environment, pre-encounter preparation, and crisis de-escalation and maintaining safety. Managing the physical environment involves awareness of and management of potential safety risks, effective use of space, and responder emotional self-regulation. For example, a CBHCR may ask that pets be contained or that a person move away from an object that might be used as a weapon. CBHCRs are also aware of providing an agitated person with enough physical space to move around and not feel trapped. They are alert to signs of increasing agitation and aggression, adjust their approach accordingly, and identify exits in the event they need to disengage quickly to maintain their own safety. Pre-encounter preparation involves gathering information, coordinating with team members and communicating while enroute. Crisis de-escalation skills include verbal de-escalation, body language and use of time and space to allow the person to self-regulate. Maintaining safety also requires setting and respecting boundaries and disengaging if a situation becomes unsafe.

Two additional competency areas were noted, *Resource Navigation and Advocacy,* as well as *Self-Care*. Their appearance as additional competencies is not to imply any less importance, as both were stressed by participants and the Advisory Board as essential. *Resource Navigation and Advocacy* maintains that CBHCRs are knowledgeable about local community resources and skilled in helping people navigate complex systems to access resources such as housing, food, medical and behavioral health care, and legal services. They utilize their resource knowledge, relationships, and advocacy skills in service of the people and communities they serve. Competence in *Self-Care* requires that CBHCRs practice stress management and maintain work-life balance. They maintain boundaries and utilize supervision for support and guidance.

## Discussion

Our findings are consistent with the literature on crisis response and the lived experience of receiving crisis services. While participants were asked about and acknowledged the need for CBHCRs to have crisis assessment and intervention skills, in much of the discussion, they emphasized characteristics, values, and competencies related to engagement, rapport, and helping the person in crisis feel safe. Participants with lived experience noted that paperwork, formal assessments, and an overly diagnostic lens often prevent them from feeling as though the responder is there to help. Both emotional and physical safety are enhanced with a compassionate, nonjudgmental, culturally humble approach that meets the person where they are at and provides them space to de-escalate and express their needs. Within that context, assessment, safety planning, and connection to resources can occur.

The competencies and skills derived from this work overlap and are consistent with the core competencies and training areas included in SAMHSA’s Draft Mobile Crisis Tool Kit (Substance Abuse and Mental Health Services Administration, 2025b), which also focuses on trauma-informed, person-centered, and culturally responsive practices. The newly released guidelines for a coordinated system of crisis care (Substance Abuse and Mental Health Services Administration, 2025a) and the draft toolkit suggest that an advanced clinical credential or license is necessary for frontline crisis response, either in person or virtually for the entire interaction. A master’s degree and licensure does not guarantee the skills needed for crisis response, however. Furthermore, this guideline is not consistent with the practices in many communities that are utilizing bachelor’s-level crisis workers supervised by licensed clinicians, nor is it practical given the known workforce shortages in crisis services. As an alternative, with a competency-based approach, we maintain that it is possible to develop a training and credentialing pathway for bachelor’s-level and non-degreed individuals with diverse backgrounds that ensures a workforce with the necessary skills for crisis response in the community. This is consistent with the workforce expectations of EMTs and paramedics, who work under the oversight of an emergency medical services medical director. This would allow for more efficient use of licensed clinicians as supervisors of CBHCRs rather than as frontline responders. Some 911-dispatched community responder programs have already demonstrated that it is possible to train non-degreed responders to effectively do this work (Beck et al., 2022) and divert some behavioral health and other types of calls away from law enforcement, freeing them up for other policing activities.

The goal of this work is not to create a new model of response to behavioral health crisis, per se, or to replace existing responders. Rather, our goal is to support workforce competency and capacity for alternative/community response and mobile crisis teams. These teams may sit within the mental health or public safety systems. By creating a pathway to crisis response work for non-degreed and degreed professionals, we can expand and diversify the workforce pool, while ensuring competency needed for the work. The recognition of CBHCR as a professional identity and role may also help attract and retain people well-suited for this work.

While we did not explicitly ask advisory board members or participants about the role of CBHCRs in handling behavioral health crises that involve criminal behavior, some participants, particularly those working on community/alternative response teams, discussed handling situations that included what could be defined as low level crimes such as trespassing or disorderly conduct. The CBHCR role as envisioned by our team, the advisory board and many of our participants, does not include enforcing laws or arrest authority. Rather, CBHCRs responding to crisis situations involving low level nonviolent crimes seek to resolve such situations without engaging law enforcement. Responding to situations involving serious crimes is not within the scope of the CBHCR role, as they have no law enforcement authority. Likewise, handling calls involving significant safety risks (violent and threatening behavior, presence of a weapon) without law enforcement support is outside of the CBHCR scope. Many community/alternative response and mobile crisis team programs have successfully implemented triage protocols to determine if it is safe for teams to respond or if law enforcement support is needed. Several participants also discussed training responders to continuously assess safety and dis-engage if situations escalate beyond the team’s ability to maintain safety.

It is important to note one topic that emerged repeatedly during our advisory board meetings: the question of whether CBHCRs should be involved in involuntary interventions. While there was consensus on the value of autonomy for all people receiving crisis intervention services, there was not agreement on whether CBHCRs should provide consent-based services only, or whether they should ever be involved in initiating or providing any involuntary interventions. Some participants felt strongly that CBHCRs should only provide consent-based services and never be involved in initiating involuntary interventions. Other participants thought there could be value in having a CBHCR with the values, competencies, and skills described here participate in the process of involuntary interventions should they be required (while acknowledging they should be used as a last resort). In reality, whether or not a CBHCR participates in these decisions and processes will likely be dictated by the type of team CBHCRs are part of, state statutes around involuntary interventions, and the available resources in the community.

We must acknowledge that while we worked to recruit diverse perspectives for our advisory board, key informant interviews and focus groups, we may have missed important perspectives on the values, competencies, and skills needed for effective response to behavioral health crises in the community. That the themes were relatively consistent across the literature review, advisory board, interviews, and focus groups gives us some assurance. However, there may be perspectives inadvertently excluded. We will continue to seek input from new sources as this work continues.

## Conclusion

The goal of the work described in this article is to create the foundation for a recognized professional workforce of CBHCRs that has the professional values, competencies, and skills necessary to safely and effectively provide intervention and support to people experiencing mental/behavioral health crisis, as well as those with unmet social service needs that would otherwise result in a police encounter. We envision these professionals working on 911-dispatched alternative/community response teams as well as mental health system-based mobile crisis teams. We are now in the process of developing a training framework and examining the facilitators and barriers to the development of a professional credential based on the completion of training supporting the identified values, competencies and skills; supervised work experience; and a credentialling exam. We envision eligibility for this training and credentialling process being open to people with high school/GED-level education, as well as bachelor’s and graduate degrees, similar to Alcohol and Drug Counselor (ADC) certification (International Certification and Reciprocity Consortium, n.d.), with multiple levels of certification allowing for career advancement. This will support alternative/community responder programs by creating a framework and process for training and credentialling their team members. Likewise, it will provide mobile crisis teams with a skilled frontline workforce that can be supported by licensed clinicians, who can therefore be utilized more efficiently. Importantly, it will expand opportunities for crisis response workforce entry to a more diverse pool of people who are well-suited for this critical work and provide them with a professional identity and career path.

## Data Availability

Data is not currently publicly available for sharing. Those wishing to access de-identified data should contact the first author (acwatson@wayne.edu) to discuss a data use agreement.
